# First comprehensive proteome analysis of lysine crotonylation in seedling leaves of *Nicotiana tabacum*

**DOI:** 10.1038/s41598-017-03369-6

**Published:** 2017-06-07

**Authors:** Hangjun Sun, Xiaowei Liu, Fangfang Li, Wei Li, Jing Zhang, Zhixin Xiao, Lili Shen, Ying Li, Fenglong Wang, Jinguang Yang

**Affiliations:** 1 Key Laboratory of Tobacco Pest Monitoring Controlling & Integrated Management, Tobacco Research Institute of Chinese Academy of Agricultural Sciences, Qingdao, 266101 China; 2 Baoshan Branch, Yunnan tobacco company, Baoshan, 678000 China; 3 Hongyunhonghe Tobacco (Group) Co., Ltd., Kunming, 650231 China

## Abstract

Histone crotonylation is a new lysine acylation type of post-translational modification (PTM) enriched at active gene promoters and potential enhancers in yeast and mammalian cells. However, lysine crotonylation in nonhistone proteins and plant cells has not yet been studied. In the present study, we performed a global crotonylation proteome analysis of *Nicotiana tabacum* (tobacco) using high-resolution LC-MS/MS coupled with highly sensitive immune-affinity purification. A total of 2044 lysine modification sites distributed on 637 proteins were identified, representing the most abundant lysine acylation proteome reported in the plant kingdom. Similar to lysine acetylation and succinylation in plants, lysine crotonylation was related to multiple metabolism pathways, such as carbon metabolism, the citrate cycle, glycolysis, and the biosynthesis of amino acids. Importantly, 72 proteins participated in multiple processes of photosynthesis, and most of the enzymes involved in chlorophyll synthesis were modified through crotonylation. Numerous crotonylated proteins were implicated in the biosynthesis, folding, and degradation of proteins through the ubiquitin-proteasome system. Several crotonylated proteins related to chromatin organization are also discussed here. These data represent the first report of a global crotonylation proteome and provide a promising starting point for further functional research of crotonylation in nonhistone proteins.

## Introduction

Post-translational modification (PTM) is a covalent modification process resulting from the proteolytic cleavage or addition of a functional group to one amino acid. Thus far, more than 200 PTMs have been characterized (http://www.uniprot.org/help/post-translational_modification). These processes modulate protein functions by altering their localization, activity state and interactions with other proteins. Among all PTMs, lysine acetylation, originally identified in histones^1^, is one of the most studied PTMs. Early studies on lysine acetylation have focused on nuclear proteins, such as histones and transcriptional factors^2, 3^. These studies suggested that lysine acetylation was restricted to the nucleus^4, 5^. The discovery of lysine acetylation on tubulin and mitochondrial proteins suggested an important role for lysine acetylation in cellular biology in addition to chromatin biology^6–8^. Using high-resolution mass spectrometry, the high abundance of lysine acetylation outside the nucleus has been identified. Lysine acetylation is abundant in most metabolic pathways, such as glycolysis, gluconeogenesis, the tricarboxylic acid (TCA) cycle, and conserved in both eukaryotes and prokaryotes^9–13^. In addition to lysine acetylation, some new types of PTMs, such as malonylation and lysine succinylation, were identified using mass spectrometry combined with the affinity purification of modified peptides using antibodies directed against these modifications^14–20^. Similar to lysine acetylation, lysine malonylation and succinylation are important in regulating cellular metabolism, and both processes exist in eukaryotes and prokaryotes^21–24^.

Histone lysine crotonylation has recently been detected from yeast to humans and is primarily associated with active transcription^25^. Similar to histone acetylation, crotonylation also occurs on the ε-amino group of lysine but distinguishes itself from acetylation by its four-carbon length and planar orientation. Lysine crotonylation, but not acetylation, preferentially marks “escapee genes” during post-meiotic sex inactivation in mouse testes^26, 27^. Lysine crotonylation and acetylation sites overlap in histones and are catalysed through p300/CBP, a well-known histone acetyltransferase^28^. Moreover, Sirtuin family members SIRT1-3, well-studied histone deacetylases, remove crotonylation in a site-specific manner. SIRT3 is present in both mitochondria and nuclei and is expressed in the kidneys and metabolically active tissues^29^. These studies lead to a question that whether cytoplasmic proteins undergo lysine crotonylation, similar to acetylation, and play an important role in regulating cellular metabolism.

Reflecting their sessile feature, plants rapidly change their endogenous status to adapt to adverse environmental conditions. Compared with the regulation of transcription and translation, PTMs could trigger a much faster response, representing a major concern in plant science. However, studies of lysine acylation of the proteome in plant cells have primarily focused on acetylation and succinylation, confirmed in only a limited number of plant species, including Arabidopsis^10, 30, 31^, rice^11, 32^, wheat^33^, soybean^34^, pea^35^, grape^36^, tomato^37^, potato^38^, strawberry^39^, and *Brachypodium distachyon* L^40^. Moreover, relatively few proteins have been modified through acetylation or succinylation. In these plants, both lysine acetylation and succinylation have been implicated in the regulation of diverse metabolic processes, such as carbon metabolism, glycolysis, pyruvate metabolism, the TCA cycle, and photosynthesis^33, 37, 40^.

Common tobacco (*Nicotiana tabacum*) is a versatile model organism for fundamental biology research and biotechnology applications^41^. It is the source of the BY-2 plant cell line, which is a key tool for plant molecular research. Moreover, tobacco is also one of the most widely cultivated non-food crops worldwide. In the present study, we investigated the global lysine crotonylation proteome of tobacco using high-resolution LC-MS/MS coupled with highly sensitive immune-affinity purification. In total, we identified 2044 lysine crotonylation sites in 637 proteins. The identified crotonylated proteins, primarily localized to the chloroplast, cytosol, nucleus, and mitochondria, were primarily involved in carbon metabolism, photosynthesis, protein biosynthesis, folding, degradation, and chromatin organization. To our knowledge, this study is the first to describe lysine crotonylation in the global proteome, thereby expanding the current understanding of the effect of lysine crotonylation on nonhistone proteins.

## Results

### Detection of lysine-crotonylated proteins in tobacco leaves

**Figure 1:**
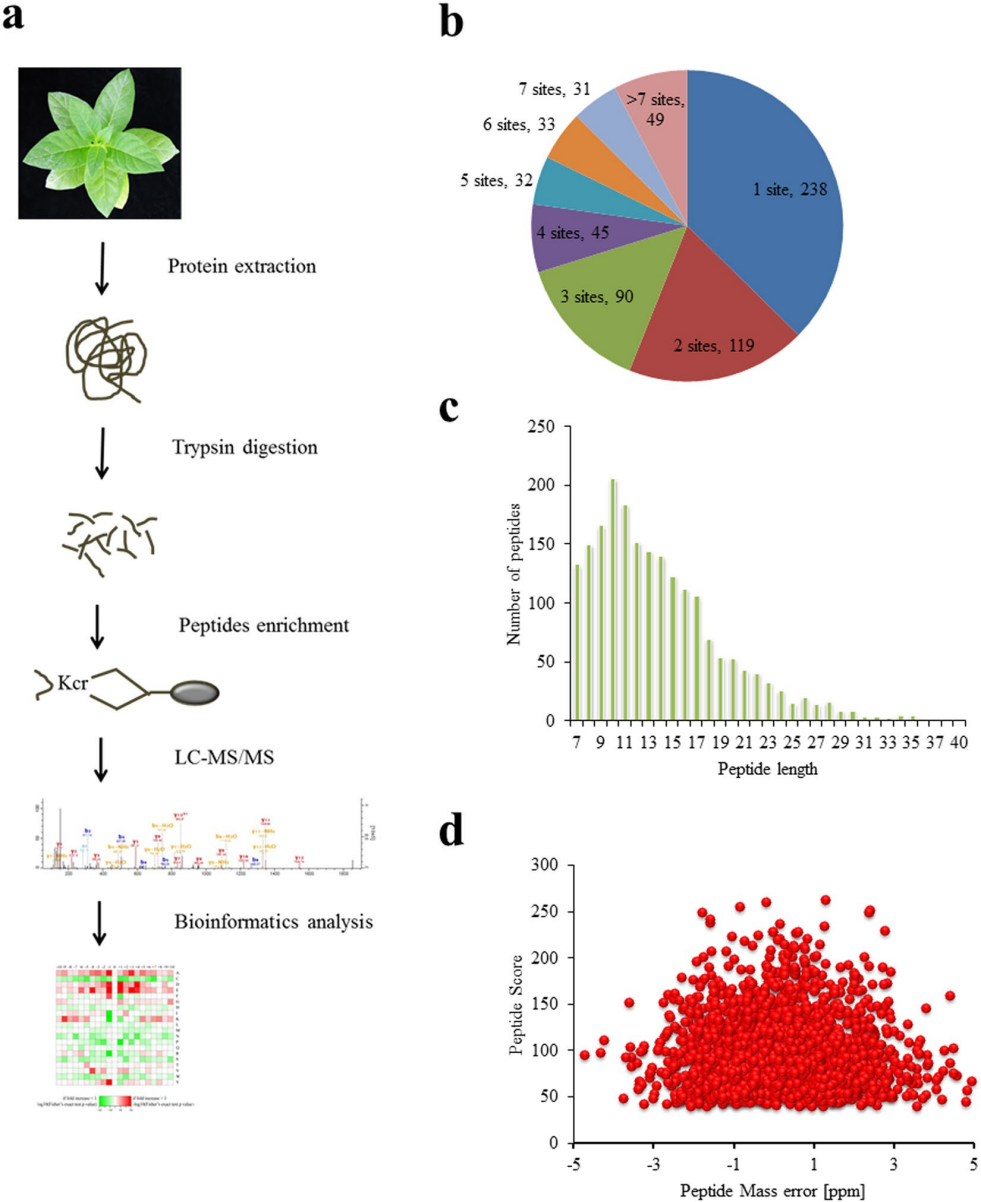
Proteome-wide identification of lysine crotonylation sites in *Nicotiana tabacum*. (**a**) Overview of experimental procedures used in the present study. Kcr indicates the crotonylated lysine. (**b**) Distribution of lysine crotonylation in one protein. (**c**) Distribution of lysine crotonylation peptides based on their length. (**d**) Mass error distribution of all crotonylated peptides.

To characterize the global crotonylation proteome of tobacco, a proteomic method based on sensitive immune-affinity purification and high-resolution LC-MS/MS was applied to identify crotonylated proteins and their modification sites in tobacco. An overview of the experimental procedures is shown in Fig. 1a. A total of 2044 lysine crotonylation sites distributed in 637 proteins were identified, representing the most abundant lysine acylation proteome reported in the plant kingdom (Table 1). MS/MS information related to these crotonylated peptides were deposited to iProX database with accession number IPX0000889000 (http://www.iprox.org). Detailed information for all identified crotonylated peptides and their corresponding proteins was shown in Supplementary Table S1, the scores for protein and peptide identification were shown in Supplementary Table S2. Among the 637 crotonylated proteins, 357 (56%) proteins contained one or two crotonylation sites, and 80 (13%) proteins had 7 or more crotonylation sites (Fig. 1b). Most peptides ranged from 7 to 28 amino acids in length, consistent with the properties of tryptic peptides (Fig. 1c). To confirm the validation of the MS data, the mass error of all identified peptides was assessed. The distribution of the mass error was near zero, and most of these proteins were less than 0.02 Da, suggesting that the mass accuracy of the MS data met the requirement (Fig. 1d).

**Table 1:** Comparison of tobacco crotonylation proteome with other published acylation proteome in plants.

Acylation	No. of acylation sites	No. of acylated proteins	Plant	References
Lysine acetylation	91	74	*Arabidopsis thaliana*	10
	699	389	rice	32
	416	277	wheat	33
	400	245	soybean	34
	664	358	pea	35
	138	97	grape	36
	35	31	potato	38
	1392	684	strawberry	39
	636	353	*Brachypodium distachyon* L	40
Lysine succinylation	665	261	rice	32
	347	202	tomato	37
	605	262	*Brachypodium distachyon* L	40
Lysine crotonylation	2044	637	tobacco	This study

### Motifs and secondary structures of lysine crotonylated peptides

**Figure 2:**
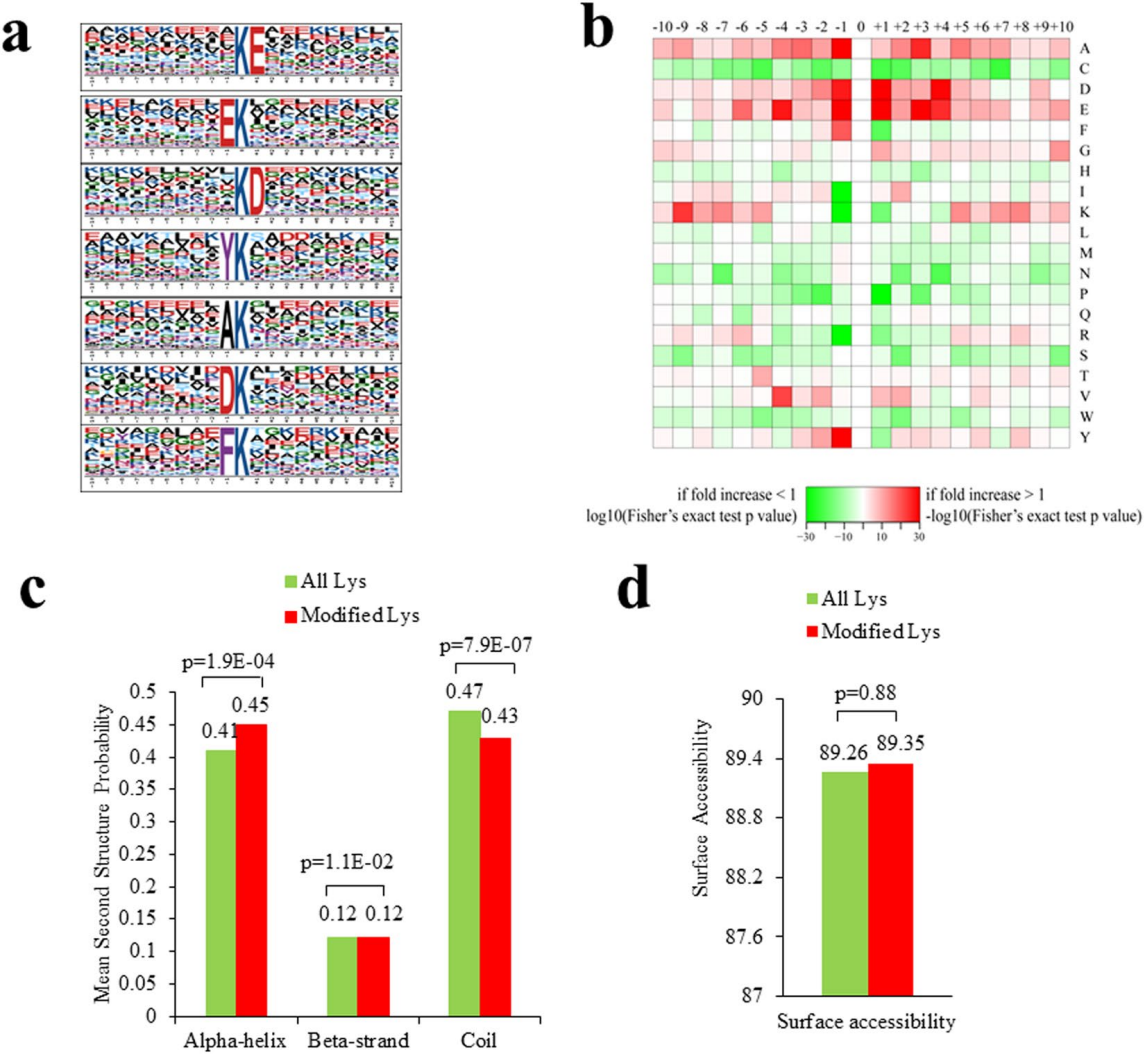
Properties of the lysine crotonylation sites. (**a**) Sequence probability logos of significantly enriched crotonylation site motifs around the lysine crotonylation sites. (**b**) Heat map of the amino acid compositions around the lysine crotonylation sites showing the frequency of different types of amino acids around this residue. Red indicates enrichment and green indicates depletion. (**c**) Probabilities of lysine crotonylation in different protein secondary structures (alpha helix, beta-strand and disordered coil). (**d**) Predicted surface accessibility of crotonylation sites.

To evaluate the nature of the crotonylated lysines in tobacco, the sequence motifs in all identified crotonylated peptides were investigated using the Motif-X programme. As shown in Supplementary Table S3, a total of nine conserved motifs were retrieved. Particularly, motifs KcrE, EKcr and KcrD (Kcr indicates the crotonylated lysine) were strikingly conserved (Fig. 2a, Supplementary Table S4). Importantly, the significantly conserved amino acids in these motifs, namely E and D, were both negatively charged, which were rarely identified in other PTMs. These motifs are likely to represent a feature of crotonylation in tobacco. Hierarchical cluster analysis was also performed to further analyse these motifs. As shown in the heat map (Fig. 2b), the enrichment of positively charged K residues was observed in the −10 to −5 and +10 to +5 positions, while negatively charged residues D and E were markedly enriched in the −4 to +4 position. Short aliphatic A residues were frequently observed in the −10 to +10 position, while the sulphur-containing C residue was not observed.

To explore the relationship between lysine crotonylation and protein secondary structures, a structural analysis of all crotonylated proteins was performed using the algorithm NetSurfP. As shown in Fig. 2c, approximately 47% of the crotonylated sites were located in α-helices, and 12% of the sites were located in β-strands. The remaining 42% of the crotonylated sites were located in disordered coils. However, considering the similarity of the distribution pattern between crotonylated lysines and all lysines, there was no tendency towards lysine crotonylation in tobacco. The surface accessibility of the crotonylated lysine sites was also evaluated. The results showed that 91% of the crotonylated lysine sites were exposed to the protein surface, close to that of all lysine residues (Fig. 2d). Therefore, lysine crotonylation likely does not affect the surface properties of modified proteins.

### Functional annotation and subcellular localization of crotonylated proteins

**Figure 3:**
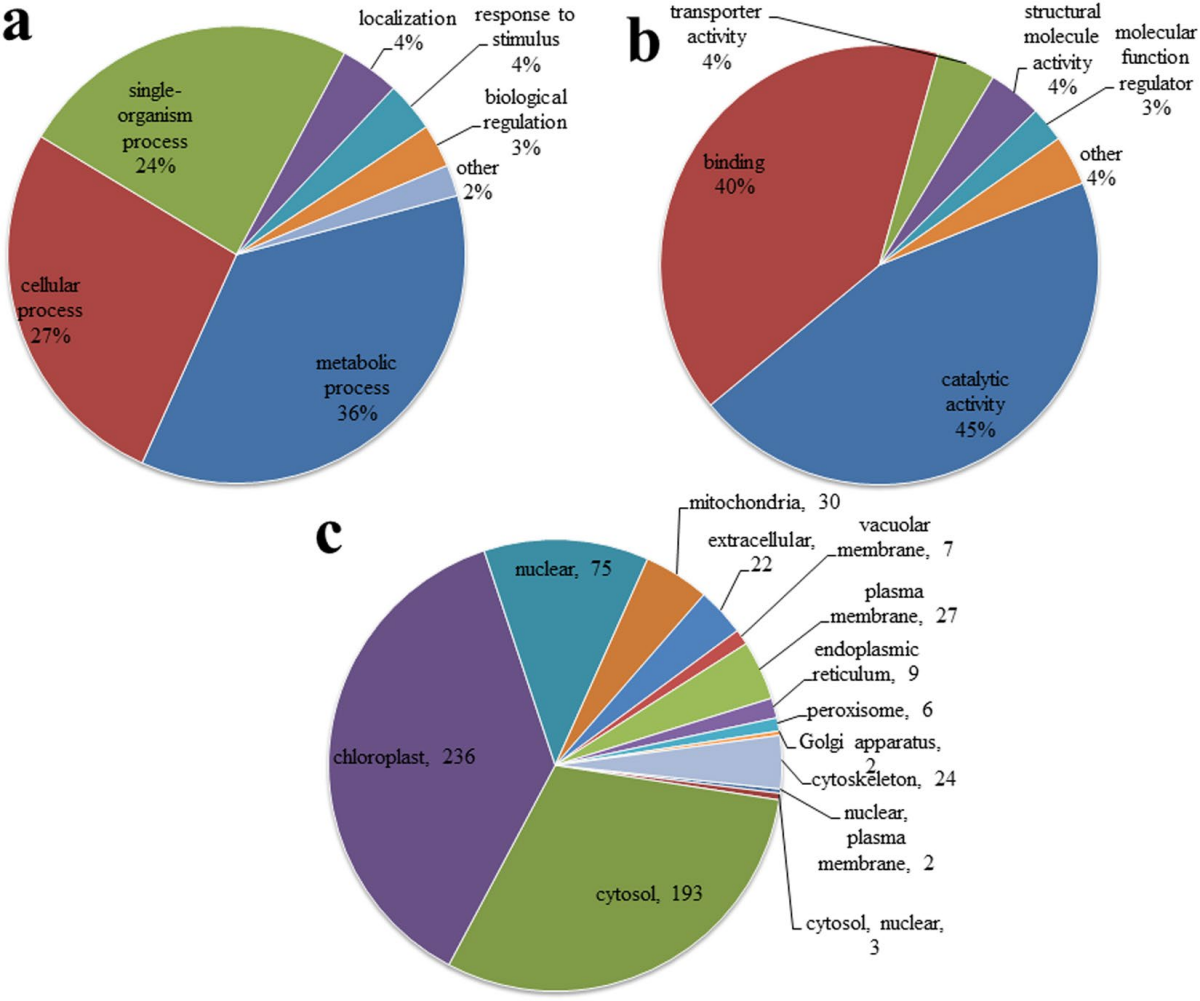
GO classification of the crotonylated proteins based on biology process (**a**) molecular functional (**b**) and subcellular localization (**c**), respectively.

To obtain an overview of the crotonylated proteins in tobacco, the Gene Ontology (GO) functional classification of all crotonylated proteins based on their biological processes, molecular functions and subcellular locations was investigated (Supplementary Table S5, Supplementary Table S6). Within the biological processes category, the majority of crotonylated proteins were related to metabolic processes, cellular processes, and single-organism processes, respectively accounting for 36, 27 and 24% of all the crotonylated proteins (Fig. 3a). For the molecular function category, 45 and 40% of the crotonylated proteins were associated with catalytic activity and binding functions, respectively (Fig. 3b). Subcellular localization analysis revealed that most of the crotonylated proteins were localized to the chloroplast (37%), cytosol (30%), nucleus (12%), and mitochondria (5%) (Fig. 3c).

### Functional enrichment analysis

**Figure 4:**
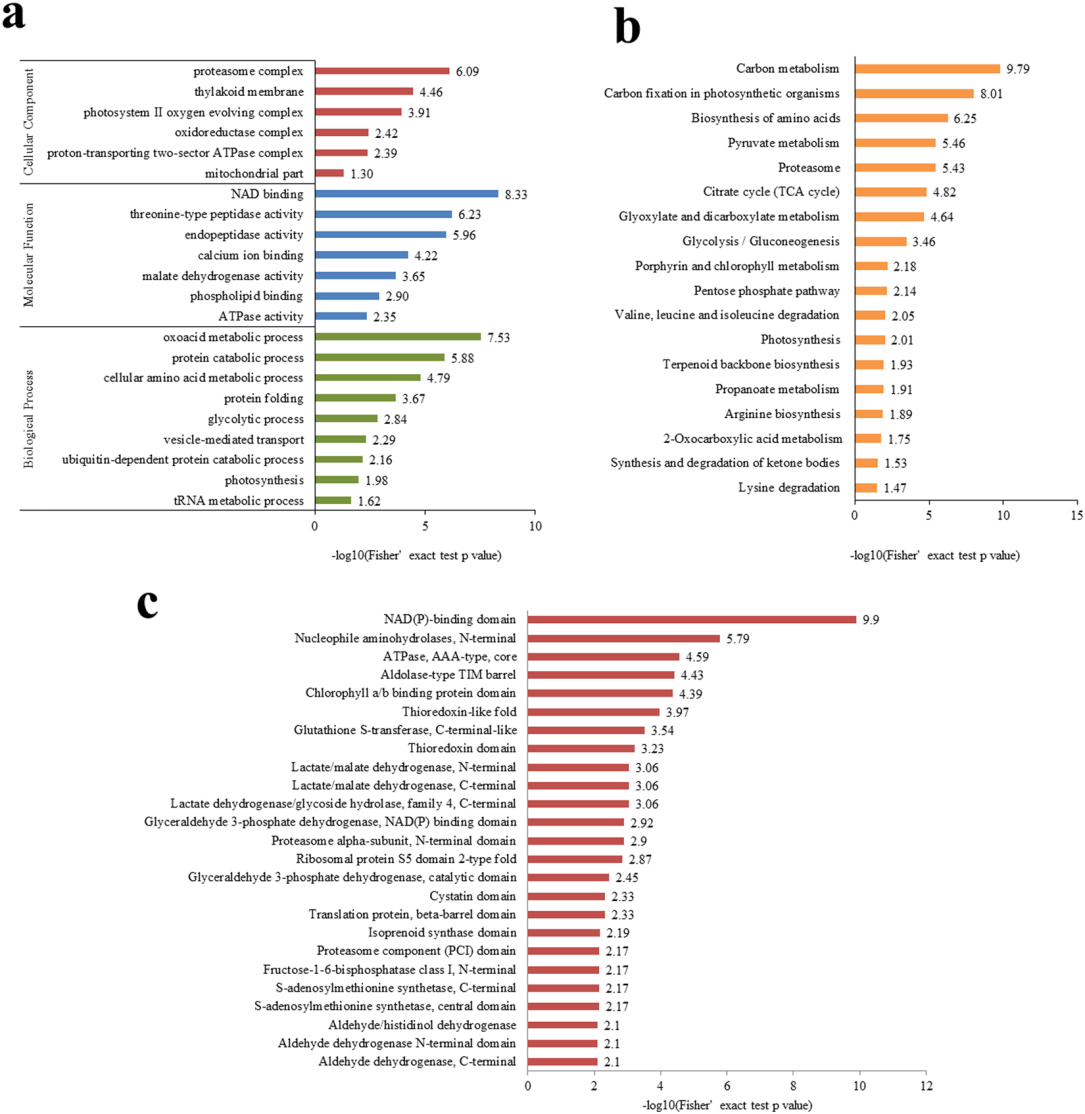
Enrichment analysis of crotonylated proteins. (**a**) GO-based enrichment analysis of crotonylated proteins in terms of cellular component, molecular function, and biological process. (**b**) KEGG pathway-based enrichment analysis. (**c**) Protein domain enrichment analysis. The numbers in X axes represent the value of significant analysis. When the value is greater than 1.3, the p value is less than 0.05, which means the data is statistically significant.

To better understand the biological function of these crotonylated proteins, we performed an enrichment analysis of the GO (Supplementary Table S7), Kyoto Encyclopedia of Genes and Genomes (KEGG) pathway (Supplementary Table S8), and Pfam domain databases (Supplementary Table S9). The enrichment analysis of the cellular components revealed that the crotonylated proteins were significantly enriched in the proteasome complex, thylakoid membrane, and photosystem II oxygen evolving complex (Fig. 4a). Based on the enrichment results of the molecular function category, most crotonylated proteins were related to NAD binding, threonine-type peptidase activity, endopeptidase activity, and calcium ion binding (Fig. 4a). In the biological processes category, most of the crotonylated proteins were implicated in oxoacid metabolic processes, protein catabolic processes, cellular amino acid metabolic processes, protein folding, ubiquitin-dependent protein catabolic processes, and photosynthesis (Fig. 4a). The KEGG pathway enrichment analysis showed that a majority of the crotonylated proteins were related to carbon metabolism, carbon fixation in photosynthetic organisms, pyruvate metabolism, proteasome, amino acid biosynthesis, the citrate cycle, glycolysis, porphyrin and chlorophyll metabolism, and photosynthesis (Fig. 4b). Consistent with these observations, Pfam domains, including the NAD(P)-binding domain, ATPase core domain, chlorophyll a/b binding protein domain, aldolase-type TIM barrel, and thioredoxin domain, were significantly enriched in crotonylated proteins (Fig. 4c), implying an important role for lysine crotonylation in these processes.

### Crotonylated proteins involved in photosynthesis

Notably, 72 crotonylated proteins were implicated in photosynthesis processes, such as light harvesting, the electron transport chain, ATP synthesis and carbon fixation (Table 2). Significantly, 73% (8/11) of the enzymes in the Calvin cycle^42^, including ribulose-1,5-bisphosphate carboxylase/oxygenase (Rubisco), phosphoglycerate kinase, glyceraldehyde-3-phosphate dehydrogenase, triose phosphate isomerase, fructose-1,6-bisphosphate aldolase, fructose-1,6-bisphosphatase, transketolase, and sedoheptulose-1,7-bisphosphatase, were extensively crotonylated at multiple sites. Among these proteins, Rubisco and phosphoglycerate kinase were crotonylated at 15 and 16 lysine sites, respectively (Table 2, Supplementary Table S1). According to the annotation in UniProt, the 15 crotonylated lysines in Rubisco were distributed around substrate binding sites (Supplementary Fig. S1a). Strikingly, the catalytic sites K201 and key amino acid residues K201 and K334 were precisely crotonylated. The same phenomenon was also observed on phosphoglycerate kinase, whose substrate-binding site and ATP binding site were surrounded with crotonylated lysines (Supplementary Fig. S1b). These results indicated that lysine crotonylation might change enzyme activity, thereby regulating photosynthesis. Moreover, most of the proteins that participated in the synthesis of chlorophyll, including glutamyl-tRNA reductase (HEMA), glutamate-1-semialdehyde 2,1-aminomutase (HEML), 5-aminolevulinate dehydratase (HEMB), uroporphyrinogen III decarboxylase (HEME), coproporphyrinogen III oxidase (HEMF), protoporphyrinogen oxidase (HEMY), magnesium chelatase, magnesium proto IX methyltransferase (CHLM), Mg-protoporphyrin IX monomethylester cyclase (CRD1), 3,8-Divinyl protochlorophyllide a 8-vinyl reductase (DVR), and protochlorophyllide oxidoreductase, were also modified by crotonyl groups (Supplementary Table S1).

**Table 2:** Crotonylated proteins involved in photosynthesis pathway.

	Protein	Protein name	Protein	Protein name
Antenna proteins	Q40481	Chlorophyll a-b binding protein	P27493	Chlorophyll a-b binding protein 21
	Q6RUN3	Chlorophyll a-b binding protein	P27495	Chlorophyll a-b binding protein 40
	Q0PWS7	Chlorophyll a-b binding protein	Q0PWS6	Chlorophyll a-b binding protein
	Q40512	Chlorophyll a-b binding protein	Q84TM7	Chlorophyll a-b binding protein
	Q0PWS5	Chlorophyll a-b binding protein	Q5DNZ6	Chlorophyll a-b binding protein
Photosystems II complex	A0A140G1Q8	Photosystem II CP43 reaction center protein	P12133	NAD(P)H-quinone oxidoreductase subunit H
	Q04126	photosystem II oxygen-evolving complex	Q40459	Oxygen-evolving enhancer protein 1
	Q9SMB4	Photosystem II 22 kDa protein	Q7DM39	Oxygen-evolving enhancer protein 2-1
	P06411	Photosystem II CP47 reaction center protein	P18212	Oxygen-evolving enhancer protein 2-2
	P69686	Photosystem II D2 protein	Q04127	Oxygen-evolving enhancer protein 2-3
	Q40519	Photosystem II 10 kDa polypeptide	Q5EFR4	oxygen-evolving protein 16 kDa subunit
	Q84QE8	Oxygen evolving complex	Q53UI6	PsbQ
Cytochrome b6f complex	P06449	Cytochrome f	P06247	Cytochrome b6
	Q02585	Cytochrome b6-f complex iron-sulfur subunit 2	P06249	Cytochrome b6-f complex subunit 4
Photosystems I complex	Q84QE7	Putative photosystem I subunit III	P06405	Photosystem I P700 chlorophyll a apoprotein A1
	Q84QE6	Photosystem I reaction center subunit X psaK	P06407	Photosystem I P700 chlorophyll a apoprotein A2
	P62094	Photosystem I iron-sulfur center	Q9T2H8	19.3 kDa photosystem I PSAD protein
	D2K7Z2	Photosystem I reaction center subunit	P35477	Plastocyanin B’/B”
Ferredoxin–NADP reductase	O04397	Ferredoxin–NADP reductase	O04977	Ferredoxin–NADP reductase
ATP synthesis complex	A0A140G1S2	ATP synthase subunit beta	P06286	ATP synthase subunit c
	W8SRJ3	ATP synthase subunit beta	P06290	ATP synthase subunit b
	Q5M9V4	ATP synthase subunit alpha	P29790	ATP synthase gamma chain
	P00823	ATP synthase subunit alpha	P32980	ATP synthase delta chain
	P00834	ATP synthase epsilon chain		
Carbon fixation	P00876	Ribulose bisphosphate carboxylase large chain	Q006P9	Malic enzyme
	A0A075M9F5	Ribulose bisphosphate carboxylase small chain	A0A077DCL8	Phosphoenolpyruvate carboxykinase
	Q42961	Phosphoglycerate kinase	A0A076KWG2	Malate dehydrogenase
	P09043	Glyceraldehyde-3-phosphate dehydrogenase A	Q9XQP4	NAD-malate dehydrogenase
	P09044	Glyceraldehyde-3-phosphate dehydrogenase B	P27154	Phosphoenolpyruvate carboxylase
	A0A068JFR6	Triosephosphate isomerase	A0A068JCD2	Chloroplast fructose-1,6-bisphosphatase
	A0A068JD04	Fructose-bisphosphate aldolase	A0A075F1V0	Malate dehydrogenase
	A0A068JIB0	Fructose-bisphosphate aldolase	Q006Q0	Malic enzyme
	F2VJ75	Fructose-bisphosphate aldolase	Q9FSF0	Malate dehydrogenase
	A0A068JD95	Fructose-1,6-bisphosphatase	A0A0K2GP10	Glyceraldehyde-3-phosphate dehydrogenase
	C3RXI5	Plastid transketolase	P09094	Glyceraldehyde-3-phosphate dehydrogenase
	A0A076KWG9	Chloroplast sedoheptulose-1,7-bisphosphatase	Q42962	Phosphoglycerate kinase
	A0A075EZS4	Glyoxisomal malate dehydrogenase		

### Crotonylated proteins involved in protein biosynthesis, folding, ubiquitin-dependent degradation

A total of 47 crotonylated proteins were identified as ribosomal proteins, translation initiation factors, elongation factors, EF-1-alpha-related GTP-binding proteins and aminoacyl-tRNA synthetases (Table 3, Supplementary Table S1), suggesting that lysine crotonylation may be involved in protein biosynthesis. Several lysine residues of HSP70 (HEAT SHOCK 70 PROTEIN), HSP90, ER-resident molecular chaperone BiP 4 (luminal-binding protein 4), and BiP 5, were moddifie by crotonyl groups (Table 3, Supplementary Table S1). These proteins assist in protein folding to avoid abnormal folding and aggregation. Ubiquitin and related proteins, such as ubiquitin extension protein, ubiquitin-conjugating enzyme, and ubiquitin activating enzyme, were also crotonylated (Table 3). Moreover, 14 proteasome subunits, which participated in ubiquitin-dependent protein degradation, were modified through crotonylation (Table 3).

**Table 3:** Crotonylated proteins involved in protein biosynthesis, folding, Ubiquitin-dependent degradation.

	Protein	Protein name	Protein	Protein name
Ribosome subunits	P06379	50S ribosomal protein L2, chloroplastic	P06374	30S ribosomal protein S16, chloroplastic
	O80361	50S ribosomal protein L4, chloroplastic	P69660	30S ribosomal protein S18, chloroplastic
	O80362	50S ribosomal protein L10, chloroplastic	P69660	30S ribosomal protein S18, chloroplastic
	P06382	50S ribosomal protein L14, chloroplastic	P25998	60S ribosomal protein L8
	P06386	50S ribosomal protein L20, chloroplastic	A0A0D3QSL6	60S ribosomal protein L17
	P06391	50S ribosomal protein L23, chloroplastic	Q07761	60S ribosomal protein L23a
	P30956	50S ribosomal protein L28, chloroplastic	Q285L8	40S ribosomal protein S3a
	P30956	50S ribosomal protein L28, chloroplastic	P29345	40S ribosomal protein S6 (Fragment)
	P02376	30S ribosomal protein S19, chloroplastic	A0A077D9P0	40S ribosomal protein S17-like protein
	P06355	30S ribosomal protein S2, chloroplastic	Q6TKQ9	Ribosomal protein L3B
	P06357	30S ribosomal protein S3, chloroplastic	Q6TKR0	Ribosomal protein L3A
	P06359	30S ribosomal protein S4, chloroplastic	Q9FSF6	Ribosomal protein L11-like (Fragment)
	P62732	30S ribosomal protein S7, chloroplastic	A0A076L4N7	Cytoplasmic ribosomal protein S13
	P62129	30S ribosomal protein S12, chloroplastic	A0A076L2E2	Ribosomal protein S25
	P06373	30S ribosomal protein S15, chloroplastic		
Translation initiation factors	Q40554	Eukaryotic translation initiation factor 3 subunit A	A0A075EYQ6	Eukaryotic translation initiation factor 5A
	P56821	Eukaryotic translation initiation factor 3 subunit B	A0A077D849	Eukaryotic translation initiation factor 5A
	Q40471	Eukaryotic initiation factor 4A-9	A0A075QVP3	Eukaryotic translation initiation factor NCBP-like protein
	A0A075QPA9	Eukaryotic initiation factor iso4E	A0A075EYP9	Translation initiation factor IF1
Elongation factors	P93769	Elongation factor 1-alpha	Q9FEL2	Elongation factor 2
	Q40581	EF-1-alpha-related GTP-binding protein	Q9FEL3	Elongation factor 2
	A0A077DCL2	Elongation factor 1-delta-like isoform 2	P68158	Elongation factor Tu, chloroplastic
	P93352	Elongation factor 2		
Aminoacyl-tRNA synthetases	A0A077D7Q3	Cytoplasmic asparagine-tRNA ligase 1	Q43794	Glutamate–tRNA ligase, chloroplastic/mitochondrial
	Q9FEL1	Lysyl-tRNA synthetase		
Molecular chaperones	Q03684	Luminal-binding protein 4	I7GVS5	Heat shock protein 70
	Q03685	Luminal-binding protein 5	Q67BD0	Heat shock protein 70-3
	G9MD86	Heat shock protein 90	P36182	Heat shock protein 82
	G9MD87	Heat shock protein 90	Q9ZT13	101 kDa heat shock protein
	Q14TB1	Heat shock protein 90		
Ubiquitin	A0A075F2H4	Ubiquitin-conjugating enzyme E2 36-like protein	Q40578	Ubiquinol oxidase 2, mitochondrial
	B6A8D0	Ubiquitin	Q45FL8	Ubiquitin extension protein
	B6V765	Ubiquitin specific protease 12	Q5M9U1	NADH-ubiquinone oxidoreductase chain 6
	O49905	Polyubiquitin	Q75VJ8	Ubiquitin activating enzyme 2
Proteasome subunits	L7UU40	26S proteasome ATPase regulatory subunit 6	Q93X34	Proteasome subunit alpha type
Proteasome subunits	P93395	Proteasome subunit beta type-6	Q93X35	Proteasome subunit alpha type
	P93768	Probable 26S proteasome non-ATPase regulatory subunit 3	Q93X37	Putative alpha5 proteasome subunit
	Q93X30	Proteasome subunit beta type	Q93X38	Putative alpha4 proteasome subunit
	Q93X31	Putative beta5 proteasome subunit	Q93X39	Putative alpha3 proteasome subunit
	Q93X32	Putative beta4 proteasome subunit	Q9XG77	Proteasome subunit alpha type-6
	Q93X33	Putative beta 3 proteasome subunit	Q9XGH8	Putative preprocysteine proteinase

### Protein interaction network of the crotonylated proteins in tobacco

**Figure 5:**
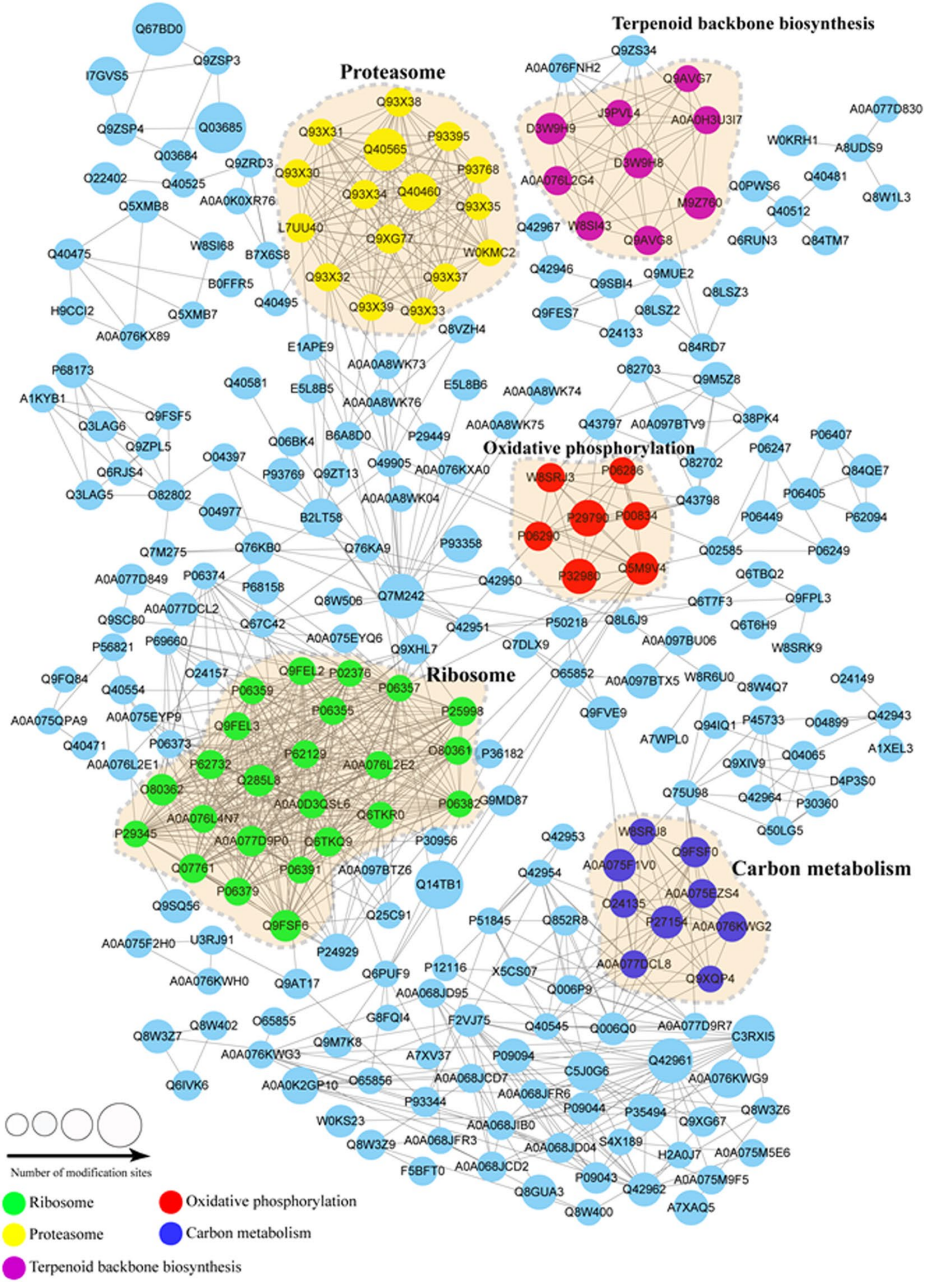
Interaction networks of the crotonylated proteins in tobacco.

To further identify the cellular processes regulated through crotonylation in tobacco, the crotonylated protein interaction network was established using an algorithm in Cytoscape software. A total of 264 acetylated proteins were mapped to the protein interaction database (Supplementary Table S10), presenting a global view of the diverse cellular functions of crotonylated proteins in tobacco. As shown in Fig. 5, crotonylated protein involved in ribosome, proteasome, carbon metabolism, oxidative phosphorylation, and terpenoid backbone biosynthesis were retrieved, comprising a dense protein interaction network. The physiological interactions among these crotonylated protein complexes likely contribute to their cooperation and coordination in tobacco.

## Discussion

Histone crotonylation is a new lysine acylation type of PTM enriched at active gene promoters and potential enhancers in mammalian cells^25^. Crotonylation is catalysed through histone acetyltransferase p300/CBP^28^, ‘read’ by YEATS2 and AF9, ‘erased’ by Sirtuin family members SIRT1-3 in yeast and mammals^29, 43–46^. However, the lysine crotonylation of nonhistone proteins and in plant cells has not yet been studied. To determine whether lysine crotonylation also exists in plants and to study its function in cellular processes, a global crotonylation tobacco proteome was realized using high-resolution LC-MS/MS coupled with highly sensitive immune-affinity purification. A total of 2044 lysine crotonylation sites distributed in 637 proteins were identified, representing the most abundant lysine acylation proteome reported in the plant kingdom. These crotonylated proteins were associated with diverse biological processes, including multiple metabolic pathways, chromatin organization, protein biosynthesis, folding, and degradation. The protein interaction network analysis also suggested that a wide range of interactions involved in these biological processes was likely modulated through protein crotonylation.

Carbon is one of the most important macroelements, providing the backbone for biological macromolecules. Lysine acetylation and succinylation in plants have been implicated in carbon metabolism, glycolysis, pyruvate metabolism, TCA cycle, pentose phosphate pathway, glyoxylate and dicarboxylate metabolism^32, 33, 37, 40^. The results of the present study showed that numerous enzymes in these metabolism pathways were also modified through crotonylation. In plants, one of the most important metabolic processes is photosynthesis. In the present study, there are 236 crotonylated proteins were localized to the chloroplast. Among these proteins, a total of 72 proteins were involved in photosynthesis processes. For example, 10, 14, 4, 8, 2, 9, and 25 proteins, identified as members of antenna proteins, photosystems II complex, cytochrome b6f complex, photosystems I complex, ferredoxin-NADP reductase, ATP synthesis complex, and the carbon fixation pathway, respectively. Significantly, 73% (8/11) enzymes in the Calvin cycle^42^ were extensively crotonylated at multiple sites, with an average of 10. For example, ribulose-1,5-bisphosphate carboxylase/oxygenase (Rubisco), the key carbon fixation enzyme, was crotonylated at 15 amino acid sites. The key amino acid residues of Rubisco, K201 and K334 which were identified as acetylated resulting in the downregulation of Rubisco activity^47^, also modified through crotonylation. This result suggested that crotonylation might change Rubisco activity in coordination with acetylation. Moreover, the two Rubisco activase isoforms^48^, involved in the light activation of Rubisco, were also crotonylated at 24 sites. Moreover, 67% (10/15) of the enzymes involved in chlorophyll synthesis^49^ were also modified through crotonylation. To our knowledge, until recently, there have been no reports of lysine acylation in chlorophyll metabolism. These results suggested that lysine crotonylation might play a role in regulating carbon metabolism and photosynthesis.

Proteins are macromolecules that, in addition to carbohydrates, perform a vast array of functions within organisms. Proteins comprise amino acids and are synthesized through translation. In plants, proteins can be degraded in two ways - proteolysis in the vacuole or via the ubiquitin-proteasome system. The data in the present study revealed that lysine crotonylation was related to the synthesis and degradation of multiple amino acids, such as lysine, valine, leucine and isoleucine. The ribosome serves as the factory of protein synthesis. In the present study, we identified 47 crotonylated proteins associated with translation, including 29 ribosome subunits, 8 translation initiation factors, 7 elongation factors, and 3 aminoacyl-tRNA synthetases. After synthesis in the ribosome, the polypeptide chain rapidly folds into its characteristic and functional three-dimensional structure from a random coil. This process is accomplished through the assistance of chaperones, such as the ER-resident molecular chaperone BiP, the HSP70 family, and the HSP90 family^50–55^. The data in the present study showed that lysine residues in members of HSP70 and HSP90 were extensively crotonylated in tobacco. Moreover, Bip 4 and Bip 5 were also extensively modified through crotonylation, suggesting an important role for lysine crotonylation in protein folding. If several rounds of chaperone-assisted folding are futile, unfolded or misfolded proteins are recognized and targeted by ubiquitin and subsequently degraded by proteasomes^56, 57^. In the present study, we found ubiquitin, ubiquitin extension protein, ubiquitin-conjugating enzyme, and ubiquitin-activating enzyme, are all modified through crotonylation. Furthermore, 14 proteasome subunits were also crotonylated. These results indicated the likely involvement of lysine crotonylation in regulating protein synthesis, folding, and ubiquitin-dependent degradation.

The organization of the eukaryotic genome into nucleosomes dramatically impacts the regulation of gene expression. The structure of the nucleosome core is relatively invariant in eukaryotic organisms, and includes a 147-bp segment of DNA and two copies of each of the four core histone proteins^58^. Histone chaperone nucleosome assembly protein 1 (Nap1) has been implicated in nucleosome assembly by eliminating competing, nonnucleosomal histone-DNA interactions^59^. The data presented here showed that tobacco histones H1, H2A, H2B, H3, and H4, and nucleosome assembly proteins Nap1;2, Nap1;3, and Nap1;4, were modified through crotonylation, indicating a potential role for lysine crotonylation in nucleosome assembly or disassembly. As complementary evidence, topoisomerase I, required for efficient nucleosome disassembly at gene promoter regions^60^, was also crotonylated in the present study. Nucleosomes are folded through a series of higher-order structures to eventually form a chromosome. An important factor in higher-order organization is the nuclear matrix, which serves as a scaffold for loops of chromatin^61^. Nuclear matrix has been proposed to play a role in regulating transcription, DNA replication, and RNA processing^62^. Chromosomal DNA was anchored to nuclear matrix by its matrix-associated regions (MARs), bound by matrix attachment region-binding protein^63^. Histone acetyltransferase (HAT) p300 and deacetylase SIRT1 interacts with matrix attachment region-binding protein SAF-A and SATB1, respectively, and thereby regulates gene expression^64, 65^. Surprisingly, in the present study, a matrix attachment region binding filament-like protein (MFP1) was identified as crotonylated at 20 amino acid sites, and even its homologue was also crotonylated at 8 amino acid sites. MFP1 is a conserved nuclear and chloroplast DNA-binding protein in plants; however, its physiological function is not understood^66–68^. Considering that p300 and SIRT1 possess crotonylation and decrotonylation activities, respectively, in animals^25, 28, 29^, it is an interesting assumption that the crotonylated or decrotonylated form of MFP1 was also associated with the regulation of gene expression. In addition to these crotonylated protein that might be associated with the assembly of nucleosome and chromatin, we identified a G-strand-specific single-stranded telomere-binding protein (GTBP), associated with maintaining telomere stability, also modified through crotonyl groups^69, 70^. These results indicated the likely involvement of lysine crotonylation in chromatin organization and gene regulation at least in tobacco.

In summary, the present study provided the first global lysine crotonylation proteome in tobacco. These data revealed lots of crotonylated proteins associated with diverse aspects of cellular process, particularly carbon metabolism, photosynthesis, protein biosynthesis, folding, degradation, and chromatin organization. These finding raised some questions that if the crotonylation of these proteins are related to biological functions and that if crotonylation changes in different situations. All these questions should be addressed in the future work. Nevertheless, the results presented here may provide a promising starting point for further functional research of crotonylation in nonhistone proteins.

## Materials and Methods

### Plant materials and growth conditions

Tobacco were grown in a greenhouse at 25 °C and a photoperiod of 16/8 h (light/dark). The leaves were excised from 4-week-old seedlings with three biological replicates and immediately used for protein extraction.

### Protein Extraction

The samples were grinded to powder in liquid nitrogen, and subsequently mixed with extraction buffer (8 M urea, 2 mM EDTA, 3 μM TSA, 50 mM NAM, 10 mM DTT and 1% Protease Inhibitor Cocktail, Millipore). The remaining debris was removed through centrifugation at 20,000 g for 10 min at 4 °C. Finally, the proteins were precipitated using cold 15% TCA for 2 h at −20 °C. After centrifugation at 4 °C for 10 min, the supernatant was discarded. The remaining precipitate was washed three times with cold acetone. The protein was redissolved in buffer (8 M urea, 100 mM NH_4_CO_3_, pH 8.0) and the protein concentration was determined using the 2-D Quant kit (GE Healthcare) according to the manufacturer’s instructions.

### Trypsin Digestion

For digestion, the protein solution was reduced with 10 mM DTT for 1 h at 37 °C and alkylated with 20 mM IAA for 45 min at room temperature in darkness. For trypsin digestion, the protein sample was diluted after adding 100 mM NH_4_CO_3_ to a urea concentration of less than 2 M. Finally, trypsin was added at 1:50 trypsin-to-protein mass ratio for the first digestion overnight and a 1:100 trypsin-to-protein mass ratio for a second 4-h digestion.

### HPLC Fractionation

The sample was subsequently fractionated through high pH reverse-phase HPLC using an Agilent 300 Extend C18 column (5 μm particles, 4.6 mm ID, 250 mm length). Briefly, the peptides were separated into 80 fractions using a gradient of 2% to 60% acetonitrile in 10 mM ammonium bicarbonate, pH 10, over 80 min. Subsequently, the peptides were combined into 6 fractions and dried using vacuum centrifugation.

### Affinity Enrichment

To enrich Kcro peptides, tryptic peptides dissolved in NETN buffer (100 mM NaCl, 1 mM EDTA, 50 mM Tris-HCl, and 0.5% NP-40, pH 8.0) were incubated with pre-washed antibody beads (PTM Biolabs) at 4 °C overnight with gentle shaking. The beads were washed four times with NETN buffer and twice with ddH_2_O. The bound peptides were eluted from the beads using 0.1% TFA. The eluted fractions were combined and vacuum-dried. The resulting peptides were cleaned with C18 ZipTips (Millipore) according to the manufacturer’s instructions, followed by LC-MS/MS analysis.

### Quantitative Proteomic Analysis by LC-MS/MS

The peptides were dissolved in 0.1% FA and directly loaded onto a reversed-phase pre-column (Acclaim PepMap 100, Thermo Scientific). Peptide separation was performed using a reversed-phase analytical column (Acclaim PepMap RSLC, Thermo Scientific). The gradient comprised an increase from 6% to 22% solvent B (0.1% FA in 98% ACN) for 24 min, 22% to 40% for 8 min and climbing to 80% in 5 min, subsequently holding at 80% for the last 3 min, all at a constant flow rate of 300 nl/min on an EASY-nLC 1000 UPLC system, the resulting peptides were analysed using the Q Exactive^TM^ Plus hybrid quadrupole-Orbitrap mass spectrometer (ThermoFisher Scientific). The peptides were subjected to NSI source followed by tandem mass spectrometry (MS/MS) in Q Exactive^TM^ plus (Thermo) coupled online to the UPLC. Intact peptides were detected in the Orbitrap at a resolution of 70,000. The peptides were selected for MS/MS using NCE setting as 30; ion fragments were detected using Orbitrap at a resolution of 17,500. A data-dependent procedure that alternated between one MS scan followed by 20 MS/MS scans was applied for the top 20 precursor ions above a threshold ion count of 5E3 in the MS survey scan with 15.0 s dynamic exclusion. The electrospray voltage applied was 2.0 kV. Automatic gain control (AGC) was used to prevent overfilling of the Orbitrap; 5E4 ions were accumulated for generation of MS/MS spectra. For MS scans, the m/z scan range was 350 to 1800. Fixed first mass was set as 100 m/z.

### Database Search

The resulting MS/MS data was processed using MaxQuant with integrated Andromeda search engine (v.1.5.1.8). Tandem mass spectra were searched against UniProt tobacco database concatenated with reverse decoy database. Trypsin/P was specified as cleavage enzyme allowing up to 4 missing cleavages, 5 modifications per peptide and 5 charges. Mass error was set to 10 ppm for precursor ions and 0.02 Da for fragment ions. Carbamidomethylation on Cys was specified as fixed modification and oxidation on Met, crotonylation on Lys and crotonylation on protein N-terminal were specified as variable modifications. False discovery rate (FDR) thresholds for protein, peptide and modification sites were specified at 1%. Minimum peptide length was set at 7. All the other parameters in MaxQuant were set to default values. The site localization probability was set as >0.75.

### Bioinformatics Methods

Motif-X software (http://motif-x.med.harvard.edu/) was used to analyse the model of sequences constituted with amino acids in specific positions of acetyl-21-mers (10 amino acids upstream and downstream of the site) in all protein sequences^71^. For further hierarchical clustering based on categories, all the acetylation substance categories obtained after enrichment were first collated along with their *p*-*value*s, and subsequently filtered for those categories at least enriched in one of the clusters with a *p*-value < 0.05. This filtered *p*-value matrix was transformed by the function *x* = −log (*p*-*value*), and the *x* values for each category were *z*-transformed. These *z* scores were subsequently clustered using one-way hierarchical clustering (Euclidean distance, average linkage clustering) in the Genesis programme. The cluster membership was visualized using a heat map through the “heatmap.2” function in the “gplot2” R-package. Secondary structures were predicted using NetSurfP. Gene Ontology (GO) annotation proteome was derived from the UniProt-GOA database (http://www.ebi.ac.uk/GOA/). The proteins were classified using Gene Ontology annotation based on three categories: biological process, cellular component and molecular function. The protein subcellular localization was analysed using Wolfpsort (http://www.genscript.com/wolf-psort.html). The KEGG was used to annotate protein pathways. GO term, protein domain, and KEGG pathway enrichment were performed using the DAVID bioinformatics resources 6.7. Fisher’s exact test was used to examine the enrichment or depletion (two-tailed test) of specific annotation terms among members of resulting protein clusters. Correction for multiple hypothesis testing was performed using standard false discovery rate control methods. Any terms with adjusted *p*-*values* below 0.05 in any of the clusters were treated as significant. The Search Tool for Retrieval of Interacting Genes/Proteins (STRING) database (http://string-db.org/) was used for PPI analysis. Cytoscape (version 3.0) software was used to display the network^72^.

## Electronic supplementary material

supplementary figure S1

supplementary table S1

supplementary table S2

supplementary table S3

supplementary table S4

supplementary table S5

supplementary table S6

supplementary table S7

supplementary table S8

supplementary table S9

supplementary table S10
