# Supplementary material for: First comprehensive proteome analysis of lysine crotonylation in seedling leaves of Nicotiana tabacum
Source: Sci Rep. 2017 Jun 7;7:3013. doi: 10.1038/s41598-017-03369-6 (PMC5462846; doi:10.1038/s41598-017-03369-6)
Supplement: Supplementary file 1 — supplementary figure S1 [file 41598_2017_3369_MOESM1_ESM.doc]

**First comprehensive proteome analysis of lysine crotonylation in seedling leaves of *Nicotiana tabacum***

Hangjun Sun^1^, Xiaowei Liu^1^, Fangfang Li^1^, Wei Li^2^, Jing Zhang^2^, Zhixin Xiao^3^, Lili Shen^1^, Ying Li^1^, Fenglong Wang^1,*^, Jinguang Yang^1,*^

^1^Key Laboratory of Tobacco Pest Monitoring Controlling＆Integrated Management, Tobacco Research Institute of Chinese Academy of Agricultural Sciences, Qingdao, 266101, China. ^2^Baoshan Branch, Yunnan tobacco company, Baoshan, 678000, China. ^3^ Hongyunhonghe Tobacco (Group) Co., Ltd., Kunming, 650231, China. *Correspondence should be addressed to J.Y. (yangjinguang@caas.cn) or F.W. (wangfenglong@caas.cn)


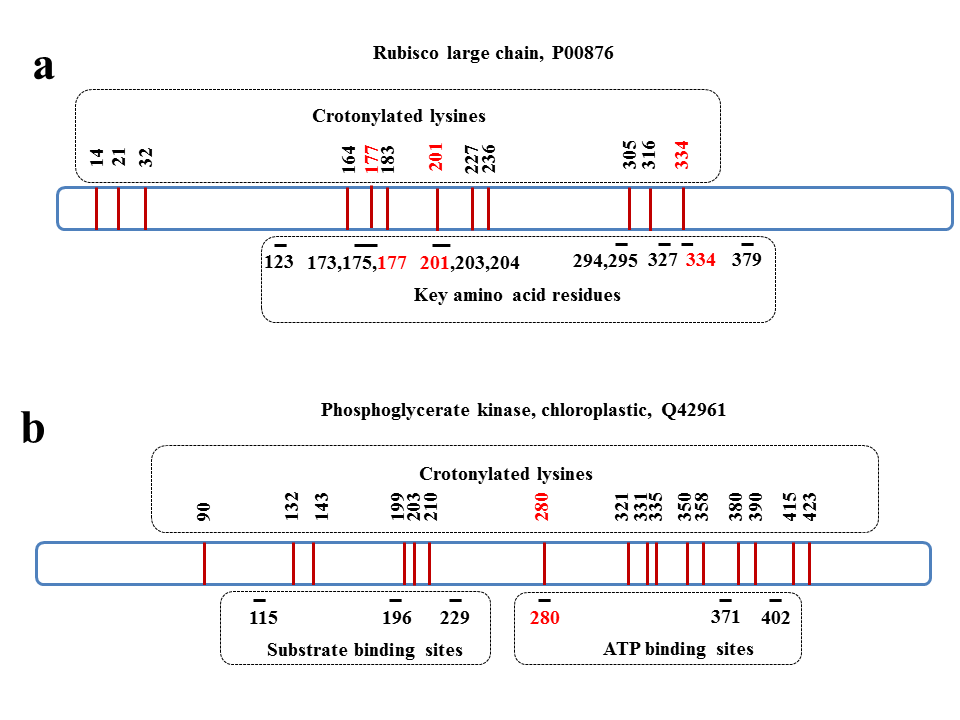


**Figure S1.** The key amino acid residues of Rubisco large chain (a) and phosphoglycerate kinase (b) were surrounded with crotonylated lysines. The numbers in red font mean the key lysine residues that modified through crotonylation.
